# The Stable Matching Problem in TBEV Enzootic Circulation: How Important Is the Perfect Tick-Virus Match?

**DOI:** 10.3390/microorganisms9010196

**Published:** 2021-01-19

**Authors:** Katrin Liebig, Mathias Boelke, Domenic Grund, Sabine Schicht, Malena Bestehorn-Willmann, Lidia Chitimia-Dobler, Gerhard Dobler, Klaus Jung, Stefanie C. Becker

**Affiliations:** 1 Institute for Parasitology, Centre for Infection Medicine, University of Veterinary Medicine Hannover, 30559 Hanover, Germany; katrin.liebig55@gmail.com (K.L.); mathias.boelke@tiho-hannover.de (M.B.); domenic.grund@gmail.com (D.G.); sabine.schicht@gmx.de (S.S.); 2 Research Center for Emerging Infections and Zoonosis, University of Veterinary Medicine Hanover, 30559 Hanover, Germany; 3 Department of Pediatric Pneumology, Allergology and Neonatology, Hannover Medical School, 30625 Hanover, Germany; 4 Bundeswehr Institute of Microbiology, Neuherbergstraße 11, 80937 Munich, Germany; Malena1BestehornWillmann@bundeswehr.org (M.B.-W.); lydiachitimia@gmail.com (L.C.-D.); gerharddobler@msn.com (G.D.); 5 Institute for Animal Breeding and Genetics, University of Veterinary Medicine Hannover, 30559 Hanover, Germany; Klaus.Jung@tiho-hannover.de

**Keywords:** tick-borne encephalitis virus, *Ixodes ricinus*, TBEV endemic focus, in vitro feeding

## Abstract

Tick-borne encephalitis virus (TBEV), like other arthropod-transmitted viruses, depends on specific vectors to complete its enzootic cycle. It has been long known that *Ixodes ricinus* ticks constitute the main vector for TBEV in Europe. In contrast to the wide distribution of the TBEV vector, the occurrence of TBEV transmission is focal and often restricted to a small parcel of land, whereas surrounding areas with seemingly similar habitat parameters are free of TBEV. Thus, the question arises which factors shape this focal distribution of TBEV in the natural habitat. To shed light on factors driving TBEV-focus formation, we used tick populations from two TBEV-foci in Lower Saxony and two TBEV-foci from Bavaria with their respective virus isolates as a showcase to analyze the impact of specific virus isolate-tick population relationships. Using artificial blood feeding and field-collected nymphal ticks as experimental means, our investigation showed that the probability of getting infected with the synonymous TBEV isolate as compared to the nonsynonymous TBEV isolate was elevated but significantly higher only in one of the four TBEV foci. More obviously, median viral RNA copy numbers were significantly higher in the synonymous virus–tick population pairings. These findings may present a hint for a coevolutionary adaptation of virus and tick populations.

## 1. Introduction

Arthropod-borne viruses (arboviruses) are maintained in nature by cycling between hematophagous arthropod vectors and vertebrate hosts. Most of the arboviruses belong to the *Bunyavirales*, *Flaviviridae*, *Togaviridae*, and *Reoviridae*, which all use RNA to code their genomic information. To succeed in dynamic host environments, especially in the case of arboviruses including two very distinct hosts, viruses need a high genetic plasticity. With an estimated range from 10^−3^ to 10^−5^ errors/nucleotide/round of replication, the RNA-dependent RNA-polymerase (RdRp) has a high error rate leading to a typical pool of viral sequence variations granting genetic plasticity and fast adaptation of RNA viruses [1,2]. As a member of the *Flaviviridae*, TBEV belongs to the RNA viruses. An infection with TBEV can result in an infection of the central nervous system in humans [3] and animals [4]. TBEV is distributed in many European countries [5,6] and the number of annual reported cases has steadily increased in the past years [7] making TBE one of the most severe arthropod-borne diseases in Germany.

TBEV is transmitted by *Ixodes ricinus* (Linnaeus, 1758) ticks in Central Europe including Germany. Although *I. ricinus* can be found all over the country, TBEV risk areas are mainly found in southern parts of Germany. Furthermore, TBEV risk areas are spatially localized, and fit with the concept of a natural focus. The natural focus is the central, crucial concept of Pavlovsky’s theory [8] with a pathogen circulation in nature independent of human presence and infection with the exception that the human is a dead-end host for the pathogen. TBEV natural foci are usually very small covering only 5000 square meters [9], thus the question arises which parameters define their borders. In addition to the spatial restriction, viral sequences in such TBEV-foci are stable over decades [10]. Considering the high mutation rates of RNA viruses, this is a remarkable characteristic of TBEV, indicating a selective pressure for specific genomic sequences of the virus. Almost nothing is known about the interaction of *I. ricinus* with TBEV and the factors shaping TBEV and tick population genetics in a TBEV-focus. However, coevolution of virus strain and tick population could have driven specific selection of tick and virus genetic markers. Such sequence-based differences are known to affect the outcome of an arbovirus infection and depend on a particular pairing of vector and virus genotypes [11]. In vitro experiments have shown the adaption of virus and vector by demonstrating that growth of TBEV on tick vector cell lines is 100–1000-fold higher as in nonvector cell lines [12]. Furthermore, the impact of environmental variations on ticks’ vector competence such as the microclimate [13] as well as the coincidence of host and tick population densities [14,15,16] has been demonstrated. Consequently, the outcome of infection seems to be a genotype–genotype–environment complex [17]. To understand this complex, different vector–virus interaction components such as genetic adaption of both, virus and vector, need to be investigated. To gain first insights into this relationship, we chose four virus isolates and tick populations from TBEV-foci in Germany. Two of the selected foci are located in close proximity to each other in Bavaria (Haselmühl and Heselbach) and a similar pair of foci was recently discovered in Lower Saxony (Barsinghausen-Mooshütte and Rauher Busch [18]). The genetic analysis of selected TBEV isolates from different endemic foci showed exchanges of 10 amino acids (aa) for the TBEV-foci Barsinghausen-Mooshütte and Rauher Busch and 19 aa difference for the TBEV-foci Haselmühl and Heselbach. We tested the susceptibility of the respective *I. ricinus* populations from each TBEV focus for the infection with the synonymous virus isolate or the genetically closely related nonsynonymous virus isolate to uncover potential correlations between virus isolate and infection success in different tick populations.

## 2. Materials and Methods

### 2.1. Tick Sampling and Maintenance

Questing *I. ricinus* nymphs were collected in April–June 2020 by flagging the low vegetation at different TBEV endemic foci in Lower Saxony (Barsinghausen 52°31′ N, 9°39′ E and Rauher Busch 52°53′ N, 8°87′ E) as well as in Bavaria (Haselmühl 49°41′ N, 11° 87′ E and Heselbach 49°32′ N, 12°15′ E). Nymphs of the TBEV endemic foci in Bavaria were sent to the laboratories of the Research Center for Emerging Infections and Zoonosis (University of Veterinary Medicine Hanover) in 50 mL tubes with fresh grass to maintain a humid environment. Immediately after receiving, ticks were stored at 4 °C for 3–7 days until experiments started. Ticks were retrieved from fridge half a day before starting of in vitro feeding to provide time for acclimatization. Ticks were identified by morphological classification and kept in an incubator with a CO_2_ content of 5%, a relative humidity of about 80% and a temperature of 34 °C during the in vitro feeding. After in vitro feeding, ticks were maintained for 7 days at room temperature (21 °C) with 95% relative humidity and a 16/8 light/dark photoperiod.

### 2.2. Virus Cultivation

Four different TBEV isolates of the European subtype were used for this study. Each virus isolate was obtained from ticks sampled in the respective TBEV-focus. Two strains were isolated from *I. ricinus* ticks collected in TBEV-foci in Lower Saxony [18]. The other strains of Bavarian TBEV-foci were kindly provided by the Bundeswehr Institute of Microbiology (Munich, Germany). Regarding virus passage, second passage of TBEV P51 and P19, and first passage of TBEV 303/16 and HB171 was used for in vitro infection of ticks. TBEV isolates were cultivated on A549 cells (ATCC^®^ CCL-185™). Cells were grown in MEM (Thermo Scientific, Waltham, MA, USA) containing 10% fetal bovine serum (FBS) and antibiotics (penicillin/streptomycin, Pan Biotech; Aidenbach, Germany; gentamicin/amphotericin, Thermo Scientific, Schwerte, Germany) and maintained at 37 °C under 5% CO_2_. Cells were inoculated with 100 µL aliquots of TBEV-RNA positive tick homogenate (diluted 1:10 MEM). After 1 h incubation at 37 °C and 5% CO_2_, unabsorbed virus and potential toxic substances from the tick supernatants were removed by rinsing cells three times with sterile PBS. The infected cells were overlaid with 10 mL of MEM supplemented with 2% FBS and antibiotics. Virus stock titration was performed by serial dilutions and 50% endpoint dilution according to Reed and Muench [19] and aliquots stored at −150 °C.

### 2.3. In Vitro Feeding

Artificial feeding was done as described in Liebig et al. [20]. In brief, an upper tick unit consisting of a glass tube in which one end covered with a silicone membrane is placed into a blood unit consisting of a plastic container. Each blood unit was filled with 5 mL of sterile, heparinized bovine blood (Fiebig Nährstofftechnik, Idstein, Germany) supplemented with 4 g/L D-(+)-glucose monohydrate (Sigma-Aldrich, Munich, Germany) and 1 mM adenosine triphosphate and 1 × 10^6^ PFU/mL of the respective virus strains. During artificial feeding, blood was changed twice a day with a maximum time interval of 14 h due to the low stability of TBEV in blood to ensure a constant virus titer. Ticks were left in the feeding unit for five days (day −5 to day 0) and at day 0 engorged ticks were removed from the membrane, cleaned by immersion in 1% hydrogen peroxide and PBS and transferred to fresh glass tubes for further incubation. At time of collection, most ticks were fully engorged. Ticks were then incubated for 7 days prior to PCR analysis, further referred to as day 7.

### 2.4. PCR

Seven days post infection (dpi) ticks were homogenized in 500 µL cell culture medium using stainless steel beads (3 mm) (Isometall, Pleidelsheim, Germany) and TissueLyser II (Qiagen, Hilden, Germany) at 20 Hz, 2 min, and three repetitions. Tick homogenates were clarified by centrifugation, and total RNA was extracted from 140 µL supernatant using the QIAamp Viral RNA Mini Kit (Qiagen, Hilden, Germany) according to the manufacturer’s instructions. Samples were tested for the presence of TBEV RNA by a quantitative RT-PCR (qRT-PCR) assay and TBEV-specific primers [21]. A standard curve was created using serial dilutions from TBEV RNA of Austrian Neudoerfl strain (U27495.1), RNase-free water served as a negative control. Each sample was run in duplicate, and the data were analyzed using AriaMx software version 1.5 (Agilent Technologies, Waldbronn, Germany). Ticks were considered positive if both duplicate samples tested positive.

### 2.5. Statistical Methods

TBEV positive rates between ticks from synonymous and nonsynonymous areas were compared using Fisher’s exact test and odds ratios. Virus loads between these areas were compared using the Mann–Whitney U test. All comparisons were performed separately for research areas in Lower Saxony and Bavaria within the statistics software R (v4.0.2, www.r-project.org). The significance level was set to α = 0.05 for all tests.

Furthermore, in an evaluation of the tests, we took into consideration the fact that 0.1–5% of experimental ticks originating from TBEV foci could have been (pre-) infected naturally [20]. To demonstrate to what extent pre-existing positivity in those few individuals may have influenced the results, we performed a series of simulations by allowing for 1, 2, or 3 misclassifications both in the synonymous and/or nonsynonymous data-sets. Simulations were performed for only those localities/areas that exhibited significant statistical difference. Briefly, 0, 1, 2, or 3 individuals at a time were swapped between the TBEV-positive and negative subsets both in the synonymous and in nonsynonymous data-sets, and such a modified data were subjected to the same statistical procedure as the original data. Eventually, these simulations were summarized using histograms of *p*-values and odds ratios (Figure S1).

Virus load tests were done alternatively with unaltered- and outliers-free data. The rationale behind excluding outliers is the experimental evidence that, in the ticks carrying the virus, blood-meal ingestion activates virus multiplication [22,23], and thus the (pre-) infected ticks are predisposed to the highest viral loads in our experiments. Outliers’ removal was done in a very conservative way using a boxplot method (allowing whiskers to have 1.5 times the length of the box on non-log-transformed plots). We chose this method because it requires less stringent assumptions concerning the data distribution.

## 3. Results

A total of 1458 *I. ricinus* nymphs were collected by flagging the vegetation in different TBEV endemic foci in Lower Saxony (Barsinghausen-Mooshütte and Rauher Busch) as well as two foci in Bavaria (Haselmühl and Heselbach). Nymphs collected in April 2020 (Barsinghausen-Mooshütte, *n* = 444; Rauher Busch, *n* = 500), in May 2020 (Haselmühl, *n* = 141; Heselbach, *n* =158), and in June 2020 (Haselmühl, *n* = 113; Heselbach, *n* = 102) were subjected to in vitro feeding with bovine blood spiked with 1 × 10^6^ PFU/mL of the respective TBEV isolate. The ticks from the foci Barsinghausen-Mooshütte and Rauher Busch were fed either with blood containing TBEV isolate P51 (Barsinghausen) or P19 (Rauher Busch), and ticks from the TBEV-foci Haselmühl and Heselbach were fed with blood containing either TBEV isolate 303/16 (Haselmühl) or HB171 (Heselbach). The feeding rates (number of engorged ticks divided by the total number of ticks tested) were between 20% and 32% (Table S1)

Analysis of 314 engorged nymphs for TBEV RNA revealed that 93% of tested ticks were positive for viral RNA. Maximum infection rates were observed for ticks from TBEV focus Heselbach, infected with the isolate HB171 in May (100%; 27/27) as well as in June (100%; 14/14) and for ticks from Haselmühl with the TBEV isolate 303/16 from Haselmühl in June (100%; 7/7) (Table S1). Infected ticks harbor between 9 and 4.6 × 10^7^ TBEV RNA copy numbers per tick with a median of 2.37 × 10^3^ TBEV RNA copies per tick over all groups. Highest copy numbers were found in ticks from the sampling area Barsinghausen-Mooshütte infected with the synonymous TBEV isolate from Barsinghausen P51 (4.6 × 10^7^ TBEV RNA copies/tick) and the lowest copy number of nine TBEV RNA copies per tick were found in a tick from Haselmühl infected with the nonsynonymous TBEV isolate HB171 from Heselbach.

To analyze the impact of virus isolate-tick population pairings, the infection success of TBEV isolates was correlated with the respective tick origin. This analysis was performed separately for all four TBEV foci in Lower Saxony and Bavaria. The analysis revealed an overall trend for a tick population to be more likely infected with the synonymous TBEV isolate as compared to a closely related nonsynonymous TBEV isolate. This trend was only significant for ticks from the focus Heselbach (*p* = 0.0026). The correlation in Heselbach contributes to the overall significance observed for the state Bavaria (*p* = 0.0014) (Table 1). To analyze the impact of pre-existing TBEV infections in ticks collected from natural TBEV foci, we performed simulation runs for the dataset Heselbach and Bavaria by allowing for 1–3 misclassifications (Figure S1). With misclassifications in the data from Bavaria, 78% of all *p*-values were still <0.05, all odds ratios were > 1, and 73% of lower confidence interval (CI)-limits were >1 as well. With misclassifications in the data from Heselbach, 56% of all *p*-values were still <0.05, all odds ratios were >1, and 45% of lower CI-limits were >1 as well.

**Figure 1:**
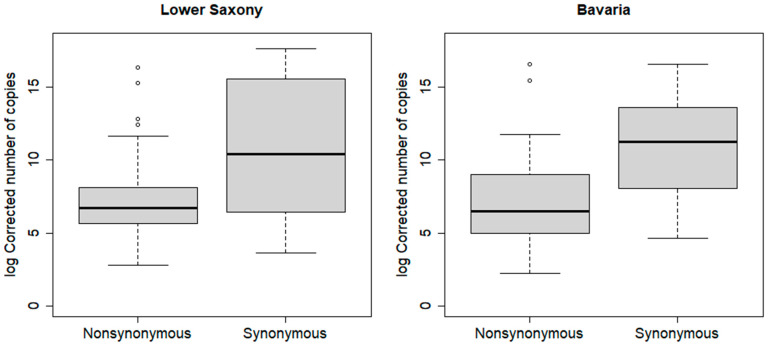
Virus loads measured in synonymous and nonsynonymous virus-tick population pairings. Outliers are represented by circles.

Next, we analyzed the efficiency of the replication of the TBEV isolates in the different tick populations and tested if replication efficiency and virus-tick population pairing are correlated. Mean viral RNA copy numbers of the synonymous and nonsynonoumous virus-tick population pairings were plotted against each other for Lower Saxony and Bavaria (Figure 1, Table 2).

Median RNA copy numbers were 842 and 679 RNA copies per tick for ticks from Lower Saxony and Bavaria, respectively, infected with the nonsynonymous TBEV isolate. In contrast, infection with the synonymous TBEV isolate led to significantly higher RNA copy numbers of 3.4 × 10^4^ (*p* < 0.01) and 7.6 × 10^4^ (*p* < 0.01) TBEV RNA copies/tick in ticks from Lower Saxony and Bavaria, respectively (Table 2). The biggest differences between nonsynonymous and synonymous were found for Haselmühl with an 872-fold higher median viral RNA copy number for the synonymous pairing compared to a nonsynonymous pairing. The weakest relationship was found for Rauher Busch in Lower Saxony with 4-fold higher median viral RNA copy numbers. However, the data of four populations analyzed separately, showed a significant result when considering all measured viral RNA copy numbers. To study the robustness of our results, 12, 17, 4, and 17 outliers were removed from the data for Barsinghausen-Mooshütte, Haselmühl, Heselbach, and Rauher Busch, respectively. A total of 44 of the outliers were removed from the synonymous units, and six from the nonsynonymous units. With this extreme process of outlier removal, Heselbach remains significant and Rauher Busch (*p* = 0.0525) and Haselmühl (*p* = 0.07) remain slightly above the significance level. Thus, we can conclude that even under very strict assumptions, evidence for higher virus loads in synonymous units is given.

## 4. Discussion

Besides mosquitoes, ticks are the most important arthropod vectors of human pathogenic diseases. In contrast to their importance, tick-virus interactions are still sparsely understood. To understand the genetic impact of TBEV isolate and tick populations for TBEV enzootic cycles in Germany, we analyzed the relationships between TBEV isolate and tick population for two different TBEV-foci in Bavaria and Lower Saxony, respectively. The TBEV-foci were located in close proximity to each other: Barsinghausen-Mooshütte versus Rauher Busch 35 km beeline and Haselmühl versus Heselbach 27 km beeline. However, the virus isolates from Lower Saxony Rauher Busch P19 and Barsinghausen P51, although being phylogenetically more closely related to each other than to other German isolates, show 10 aa exchanges [18]. A similar relationship is true for the virus isolates from Haselmühl and Heselbach, which exhibit 19 aa exchanges. This degree of diversity is on the lower level of TBEV diversity. For example, Kupča et al. [24] describe the relationship of the isolate AS33 and Salem showing 251 nucleotide differences resulting in 26 aa exchanges between those two strains. In general, TBEV sequences are conserved compared to other members of the *Flaviviridae* with only 1.8% variation based on E-gene sequences compared to 6% natural observed variation for Dengue virus, 7% for West Nile Nile-virus and 5% for Yellow fever virus (YFV) [25]. Changes in virus’s genetics, as the exchange or deletion of aa can have a strong effect on virus infection, replication, and dissemination of virus. For example, the YFV isolates YFV-17D and YFV-DAK differed in their ability to overcome the midgut barrier in *Aedes* (*Ae*.) *aegypti* mosquitoes [26] and one mutation at the position 226 on the Chikungunya virus E1 glycoprotein (E1-A226V) enhances the transmission in *Ae. albopictus* mosquitoes [27]. Regarding TBEV, Mitzel et al. [28] showed that besides the key role in host tropism the E, M, NS3, NS4A, and NS4B protein might act as viral determinants for host-specific replication. We found one aa difference between Barsinghausen-Mooshütte and Rauher Busch in the E, NS2a, and NS4b sequences, two aa difference in NS3, and five variations in the NS5 sequence, respectively [18]. Regarding the TBEV isolates Heselbach and Haselmühl, we found one aa difference in the C, E, prM, NS2b, and NS5 proteins, two differences in the NS4b and four variations in NS1, NS2a, and NS3 proteins (Bestehorn-Willmann, unpublished data). None of the variation was identical between the Bavaria and Lower Saxonian strains. However, they might still be located in the same functional domain or affect similar protein functions.

To study the impact of virus sequence differences on infection success in different tick populations, we analyzed the infection rates and TBEV RNA copy numbers for Barsinghausen-Mooshütte versus Rauher Busch and Haselmühl versus Heselbach using an artificial membrane based feeding system and nymphal stages of ticks. We chose nymphs because of two reasons, their high abundance in nature and the important role of infected adult stages for human infections. Studies have shown that infection rates of adult ticks are 5–10 times higher than in nymphs [29,30]. In addition, adult tick stages prefer larger mammalian hosts including humans.

The feeding rates in our study were highly similar between the different study groups, which was to be expected for ticks originating from sampling spots located in close proximity to each other with similar climatic conditions and habitat parameters. However, in this study ticks from Bavaria and Lower Saxony did not show different feeding rates. This observation stands in contrast to our previous study which observed significantly different feeding rates for ticks from different federal states in Germany [20]. This might be due to the reduced sampling scheme, only analyzing ticks from three months as compared to the previous study, which included two consecutive years from April to October. Furthermore, in our first study we compared a TBEV-focus (Haselmühl) with a nonendemic area (Hanover), whereas in this study we only included TBEV-foci from different federal states. Thus, it needs further clarification if ticks from TBEV-foci generally show higher feeding rates as compared to nonendemic areas.

In contrast to the moderate feeding success, the infection rates were exceptionally high in 2020. Of the 314 analyzed ticks, 93% were tested positive for TBEV RNA as compared to 38% TBEV positive samples in 2018/2019 [20]. Analysis of mean viral copy numbers also showed an increase of TBEV RNA loads as compared to our previous study with 1.40 × 10^6^ in 2020 versus 4.81 × 10^3^ TBEV RNA copies per tick over all experiments in 2018/2019, respectively. These copy numbers lie well above theoretical values derived from artificial detection of input RNA (2 × 10^2^ RNA copies per sample), indicating that the high infection rates are attributed to replication of the virus rather than residual input RNA.

Next, we analyzed if the probability of a TBEV infection after artificial feeding is linked to an adaption of TBEV isolate and tick population. To do so, we analyzed the infection rates as well as the viral RNA copy numbers of all four TBEV-foci for a favoring of synonymous virus-tick pairings over nonsynonymous pairings. Regarding the infection rates, we found only for Heselbach a significant correlation to synonymy (*p* = 0.0026), which contributes to the overall significance observed for the state Bavaria (*p* = 0.0014) (Table 1). None of the other tested TBEV foci revealed a statistically significant correlation, although we observed a trend favoring the synonymous pairing. Next, we analyzed the stability of our analysis for both, Heselbach and Bavaria. To simulate random misclassification due to pre-existing TBEV infection in ticks collected in a natural TBEV-focus, we performed simulation runs by allowing for 1, 2, or 3 misclassifications (Figure 1). We used 1–3 misclassifications based on the described TBEV infection rates in TBEV-Foci with minimal infection rates (MIR) of 0.1–5% [20] and our measured MIR for the focus Haselmühl from 2020 of 0.22% (Gerhard Dobler, unpublished results). With misclassifications in the datasets from Bavaria and Heselbach still the majority of all *p*-values were <0.05, and all odds ratios were >1. Thus, with the additional more strict assumption of misclassification, there is still some evidence for the observed correlation between virus isolate and tick population. However, this is first evidence that warrants further investigation to provide more evidence for this correlation.

To support the evidence arising from our infection data, we also compared viral RNA loads in synonymous versus nonsynonymous virus-tick populations pairings. We found significantly higher median TBEV RNA copy numbers in the synonymous pairing for both, tick populations from Lower Saxony and Bavaria (Figure 1; Table 2). Furthermore, all four TBEV foci showed a significant correlation when being analyzed separately. Again, we tested the stability of our result under the assumption of misclassifications resulting of pre-infected ticks in our experiment. Regarding viral loads, we assumed that such pre-existing infection would present themselves as outliers with exceptionally high viral RNA copy numbers. This assumption was based on a work published by Belova and colleagues [22,23] showing increased viral replication in TBEV pre-infected ticks after blood feeding. Using a very rigorous method, we removed 12, 17, 4, and 17 outliers from the data for Barsinghausen-Mooshütte, Haselmühl, Heselbach, and Rauher Busch, respectively. These numbers are well above theoretical numbers of misclassified samples, which would be maximum four ticks in Barsinghausen-Mooshütte and Rauher Busch (5% of the 84 or 91 TBEV infected ticks), three ticks in Haselmühl (5% of 61), and five ticks in Hesselbach (5% of 96). Even with this extreme process of outlier removal, Heselbach remains significant and Rauher Busch (*p* = 0.0525) and Haselmühl (*p* = 0.07) remain slightly above the significance level. Thus, we can conclude that even under very strict assumptions, evidence for higher virus loads in synonymous pairings is given. These observations could support our hypothesis of coevolution between a TBEV isolate and tick population in a TBE natural focus. Further studies are needed to support this hypothesis. Specifically, infection studies using reverse genetic systems to analyze the impact of single polymorphisms between virus isolates on the infection success and replication of TBEV in tick populations would be essential to prove such a coevolution theory. Furthermore, genetic information on tick population will be needed to analyze potential links between tick populations and virus isolates. Such studies have not been published so far for any tick-transmitted virus, but several studies describe intraspecies genetic differences and their influence on vector competence of mosquito-borne viruses. For example, *Culex pipiens* biotype *pipiens* populations in Germany show differential susceptibility for West Nile virus [31], and *Ae. albopictus* populations show population cluster specific dissemination and transmission efficiencies [32]. Similarly, *Ae. aegypti* susceptibility for dengue-2 virus is linked to yet undetected quantitative trait loci [33] and competence for chikungunya virus transmission is also dependent on mosquito genetics [34].

Factors relevant for those population-specific differences are not clear but it is rational to assume differences in intrinsic infection barriers as one cause for population specific differences in vector competence. Specifically, the midgut and salivary gland barrier may play a crucial role for the development of virus infection in the tick and the transmission of the virus by the tick. Thus far, analysis of the role of the midgut barrier has been conducted in *Amblyomma* (*A*.) *variegatum* and *Rhipicephalus* (*R*.) *appendiculatus* for *Dugbe virus* (*Nairovirus*, *Bunyavirales*). These experiments showed that infection via feeding is possible in vector ticks (*A. variegatum*) and leads to transstadial transmission of the virus, which is not the case in nonvector ticks such as *R. appendiculatus* [35]. This indicates that the midgut barrier may not only determine if an infection is established, but also block transstadial transmission of a virus. It has been shown that TBEV needs to replicate in the lining of the tick midgut where it disseminates to the hemolymph and subsequently infects other tissues reaching the highest titers in the salivary glands and reproductive organs epithelium. The higher viral RNA loads in our synonymous virus-tick population pairings might facilitate the virus escape from the midgut and the spread to other organs and thus influence the probability of the transstadial transmission of the virus, which could influence the probability of this tick to transmit TBEV to the next host.

## 5. Conclusions

In conclusion, our study provides first evidence for a virus isolate-tick population relationship that could be responsible for the focal distribution of TBEV transmission. Which genetic factors in ticks and viruses shape this relationship remains to be further investigated.

## Figures and Tables

**Table 1:** Comparison of TBEV positive rates between ticks with synonymous and nonsynonymous pairing, separately for TBEV areas Lower Saxony and Bavaria. The *p*-values were calculated using Fisher’s exact test.

TBEV Area and Focus (Tick Origin)	Pairing (Virus Isolate)	TBEV Positive Ticks (%)	TBEV Negative Ticks (%)	Odds Ratio	95%-CI	*p*-Value
Lower Saxony	Nonsynonymous	114 (93%)	8 (7%)			
	Synonymous	61 (97%)	2 (3%)	1.85	(0.33, 18.80)	0.7204
Barsinghausen	Nonsynonymous (P19)	60 (90%)	7 (10%)			
	Synonymous (P51)	24 (92%)	2 (8%)	1.18	(0.19, 12.7)	1.0000
Rauher Busch	Nonsynonymous (P51)	54 (98%)	1 (2%)			
	Synonymous (P19)	37 (100%)	0 (0%)	-	(0.02, -)	1.0000
Bavaria	Nonsynonymous	50 (82%)	11 (18%)			
	Synonymous	67 (99%)	1 (1%)	14.50 *	(2.00, 641.66)	0.0014
Haselmühl	Nonsynonymous (HB171)	35 (85%)	6 (15%)			
	Synonymous (303/16)	26 (96%)	1 (4%)	4.37	(0.48, 212.53)	0.2301
Heselbach	Nonsynonymous (303/16)	15 (75%)	5 (25%)			
	Synonymous (HB171)	41 (100%)	0 (0%)	- *	(2.15, -)	0.0026

The lines for the two Federal states are marked by grey shading; the lines showing data from the TBEV-foci within those federal states are left white. An * asterisk marks all statistically significant results.

**Table 2:** Analysis of synonymous and nonsynonymous pairing of TBEV isolates and tick population on TBEV RNA copy numbers per infected tick separately for Lower Saxony and Bavaria. The *p*-values were calculated by the Mann-Whitney *U* test.

TBEV Area and Focus (Tick Origin)	Pairing (Virus Isolate)	All Data			Outliers Removed		
		Median	Minimum; Maximum	*p*-Value	Median	Minimum; Maximum	*p*-Value
Lower Saxony	Nonsynonymous	842	16; 12,400,000	<0.01 *	781	16; 112,000	0.66
	Synonymous	34,200	38; 46,100,000		915	38; 106,000	
Barsinghausen	Nonsynonymous (P19)	2360	105; 250,000	<0.01 *	2195	105; 112,000	0.49
	Synonymous(P51)	67,950	58; 46,100,000		15,300	58; 73,300	
Rauher Busch	Nonsynonymous (P51)	376	16; 12,400,00038	<0.01 *	325	16; 107,000	0.0525
	Synonymous (P19)	1650	38; 15,800,000		907	38; 106,000	
Bavaria	Nonsynonymous	679	9; 15,500,000	<0.01 *	577	9; 128,000	<0.01 *
	Synonymous	76,100	103; 15,500,000		9995	103; 15,500,000	
Haselmühl	Nonsynonymous (HB171)	528	9; 15,500,000	<0.01 *	517	9; 128,000	0.07
	Synonymous (303/16)	437,000	103; 10,100,000		2110	103; 77,700	
Heselbach	Nonsynonymous (303/16)	1320	86; 11,900	<0.01 *	1320	86; 11,900	<0.01 *
	Synonymous (HB171)	21,100	587; 15,500,000		16,900	587; 15,500,000	

The lines for the two Federal states are marked by grey shading; the lines showing data from the TBEV-foci within those federal states are left white, An * asterisk marks all statistically significant results.

## Data Availability

The data presented in this study are available on request from the corresponding author.

## Supplementary Materials

The following are available online at https://www.mdpi.com/2076-2607/9/1/196/s1, Figure S1: Distribution of *p*-values, odds rations and lower limits of 95%-confidence intervals (CI) for the odds ratio under 1, 2 or 3 misclassifications in the comparison of synonymy and TBEV infection of ticks with respect to finding in both Bavarian sites or only in Heselbach, Table S1: Feeding and Infection rates of Ixodes ricinus ticks collected in spring 2020 and infected with TBEV.
