# Supplementary material for: The Stable Matching Problem in TBEV Enzootic Circulation: How Important Is the Perfect Tick-Virus Match?
Source: Microorganisms. 2021 Jan 19;9(1):196. doi: 10.3390/microorganisms9010196 (PMC7833397; doi:10.3390/microorganisms9010196)
Supplement: Supplementary file 1 [file microorganisms-09-00196-s001.pdf]

		2020					
		April		May		June	
Tick origin/virus isolate		Barsinghausen	Rauher Busch	Haselmühl	Heselbach	Haselmühl	Heselbach
	Feeding rate (%)	24 (105/444)	20 (101/500)	32 (45/141)	26 (41/158)	20 (23/113)	20 (20/102)
	Loss during incubation	12	9	0	0	0	0
P51 (Barsinghausen)	Infection rate (%)	92 (24/26)	98 (54/55)				
P19 (Rauher Busch)	Infection rate (%)	90 (60/67)	100 (37/37)				
303/16 (Haselmühl)	Infection rate (%)			95 (19/20)	79 (11/14)	100 (7/7)	67 (4/6)
HB171 (Heselbach)	Infection rate (%)			88 (22/25)	100 (27/27)	81 (13/16)	100 (14/14)

Supplementary Table S1: Feeding and Infection rates of *Ixodes ricinus* ticks collected in spring 2020 and infected with TBEV

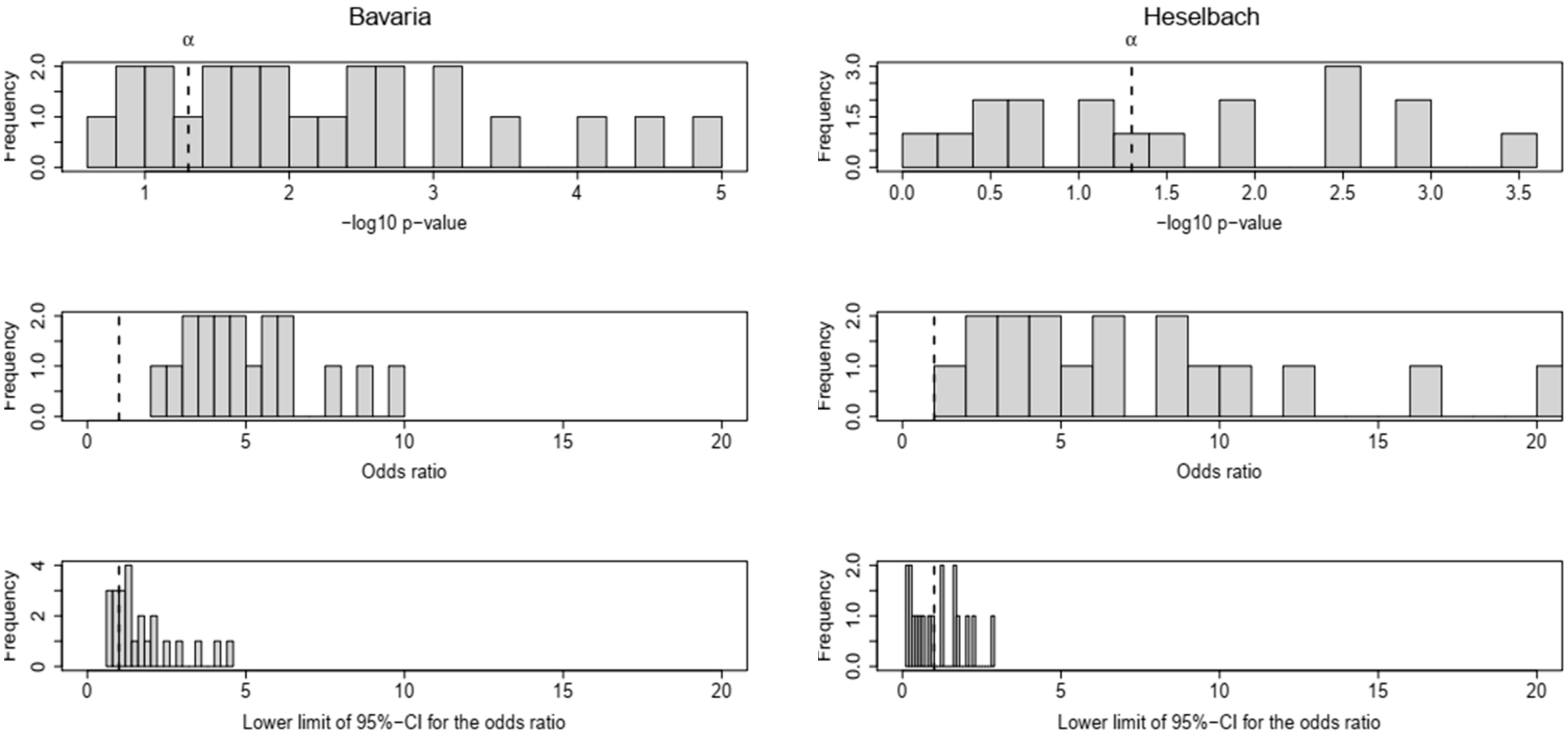

Supplementary Figure S1: Distribution of p-values, odds rations and lower limits of 95%-confidence intervals (CI) for the odds ratio under 1, 2 or 3 misclassifications in the comparison of synonymy and TBEV infection of ticks with respect to finding in both Bavarian sites or only in Heselbach.
